# "We need people to collaborate together against this disease": A qualitative exploration of perceptions of dengue fever control in caregivers' of children under 5 years, in the Peruvian Amazon

**DOI:** 10.1371/journal.pntd.0005755

**Published:** 2017-09-05

**Authors:** Amy L. Frank, Emily R. Beales, Gilles de Wildt, Graciela Meza Sanchez, Laura L. Jones

**Affiliations:** 1 University of Birmingham Medical School, University of Birmingham, Birmingham, United Kingdom; 2 Facultdad de medicina humana, Universidad de la Amazonia Peruana, Iquitos, Peru; 3 Institute of Applied Health Research, University of Birmingham, Birmingham, United Kingdom; Tulane University School of Public Health and Tropical Medicine, UNITED STATES

## Abstract

**Background:**

Dengue Fever presents a significant and growing burden of disease to endemic countries, where children are at particular risk. Worldwide, no effective anti-viral treatment has been identified, thus vector control is key for disease prevention, particularly in Peru where no vaccine is currently available. This qualitative study aimed to explore the perceptions of dengue control in caregivers’ of children under 5 years in Peru, to help direct future mosquito control programmes and strategy.

**Methods:**

Eighteen semi-structured interviews were conducted in one health centre in Iquitos, Peru. Interviews were audio-recorded, transcribed and translated by an independent translator. Data were analysed using an inductive thematic approach.

**Findings:**

Three core analytic themes were interpreted: (1) *awareness of dengue and its control*, (2) *perceived susceptibility of children*, *rural riverside communities and city inhabitants*, and (3) *perceived responsibility of vector control*. Participants were aware of dengue symptoms, transmission and larvae eradication strategies. Misconceptions about the day-time biting behaviour of the *Aedes aegypti* mosquito and confusion with other mosquito-borne diseases influenced preventative practice. Community-wide lack of cooperation was recognised as a key barrier. This was strengthened by attitudes that the government or health centre were responsible for dengue control and a belief that the disease cannot be prevented through individual actions. Participants felt powerless to prevent dengue due to assumed inevitability of infection and lack of faith in preventative practices. However, children and rural communities were believed to be most vulnerable.

**Conclusions:**

Perceptions of dengue control amongst caregivers to under 5’s were important in shaping their likelihood to participate in preventative practices. There is a need to address the perceived lack of community cooperation through strategies creating a sense of ownership of community control and enhancing social responsibility. The belief that dengue cannot be prevented by individual actions in a community also warrants attention. Specific misconceptions about dengue should be addressed through the community health worker system and further research directed to identify the needs of certain vulnerable groups.

## Author summary

Dengue fever is the most rapidly spreading mosquito-borne viral disease in the world and is a significant international health problem. It is endemic in the Peruvian Amazon and since there is no available vaccine or effective treatment, mosquito control is key. This novel study used qualitative interviews to explore the perceptions and experiences of dengue control in caregivers’ to children under 5 years, to help inform future Peruvian mosquito control programmes. Findings suggested that caregivers had basic knowledge about dengue, but that misconceptions around the mosquito itself and confusion with other diseases (like malaria) influenced people’s protective practice. Furthermore, participants felt powerless to prevent dengue since the invisible mosquito, perceived ineffective prevention methods, and rural riverside environment made the disease seem inevitable. Despite this, children and rural riverside communities were identified as most vulnerable groups. Key findings suggested a lack of community cooperation in mosquito prevention due to an attitude that the government and health centre were responsible for disease control, and the belief that dengue cannot be prevented through individual actions alone. These were important barriers to community mosquito prevention efforts. The findings from this study provide novel insights into how perceptions of dengue control can impact prevention in an endemic country.

## Introduction

Dengue fever (DF) remains the most rapidly spreading mosquito-borne viral disease in the world and it has caused more human morbidity and mortality than any other arbo-viral infection [1,2]. It currently infects 390 million people each year [3]. Dengue fever has presented an acutely difficult and progressive public health challenge in Latin America, as it has increased in prevalence, severity and spread throughout the region [4,5]. In Peru, over half the population live in areas at risk of the disease since it re-emerged in 1984 after 30 years of successful vector eradication [6]. This re-emergence has been attributed to: rapid growth and migration of urban populations, and limited resources directed towards dengue control [6]. Climate change, extreme poverty and poor water supply leading to household water storage have further compounded these effects by increasing the habitation sites of the mosquito, which now populates 17 of the 25 departments in Peru [6].

Dengue viruses are transmitted by female mosquitoes of the *Aedes* genus which are wide-spread in tropics and sub-tropics [7, 8, 9]. Dengue has been a particular health threat to children living in endemic areas [10] since young children are particularly susceptible to dengue haemorrhagic fever [1,11]. Ninety percent of cases of DHF occur in infants under 5 years of age, where there is a 2.5% risk of fatality [12]. The situation regarding a vaccine against dengue remains complex. Although a vaccine has undergone Phase III clinical trials and is recommended for use in endemic areas, it is not registered for use in young children [13]. Since no effective anti-viral treatment exists globally, and no vaccine is currently available in Peru, mosquito control is the foundation of dengue prevention nationwide [4,14]. Control strategies against the *Aedes aegypti* vector may also have the combined effect of enhancing protection from other diseases transmitted by the mosquitoes, such as zika and chikungunya. In dengue-endemic countries, control focuses on community and household mosquito eradication strategies. In the Peruvian Amazon, the city of Iquitos is endemic for DF and experiences frequent outbreaks [15]. The inaccessibility of drinking water in homes leads to the use of water storage containers [16], which provide a breeding site for the *Aedes aegypti* mosquito. In Iquitos, health promotion messages are broadcasted via radio, television and roadside billboards. These predominantly focus on symptom recognition and household eradication of breeding sites through cleaning the house and care or removal of containers that collect water (buckets, tubs, tyres for example) [17]. The local health authority further streamlines control strategies through case-by-case fumigation, larvicide water treatments, home inspections and community education [6].

According to the World Health Organisation, dengue control needs improvement to alleviate its significant burden of disease to endemic countries [18]. Further research is required to target dengue control in areas at risk. To date, few qualitative studies have explored perceptions and experiences around dengue control worldwide, but these have identified certain issues such as misconceptions, confusion with other febrile diseases, the “invisibility” of dengue and lack of responsibility in control methods [19–25]. In Iquitos, dengue-related research has concentrated on studies of epidemiology and entomology [26–28]. A recent survey evaluated community knowledge and practices [29], highlighting the misconception that *Aedes aegypti* bite during the night-time and the subsequent incorrect use of mosquito bed nets. Although informative, the nature of the survey did not allow researchers to explore in depth *why* misunderstandings exist or *how* preventative practices are influenced by perceptions. A recent qualitative study performed in Northern Peru and one Peruvian focus group study included within a WHO report found that people may consider vector control to be a lot of work, with reservations about the efficacy of interventions. [17, 30] However, as far as the authors are aware, there have been no other published qualitative studies in Peru which have explored this topic [31], highlighting an important gap in the knowledge base, particularly in this region [32,33].

This novel qualitative study sought to better understand the interrelated social and individual factors that determine perceptions, experiences and practices of dengue control, in caregivers of children under 5 years (a term which describes biological parents, step/foster/adoptive parents, wider family members and will be used from this point forward) to an at-risk group. Further understanding of caregivers’ thoughts and actions around DF can help to inform and direct future mosquito control programmes and strategy in the Peruvian Amazon.

## Methods

### Ethics statement

Ethical approval was gained from the University of Birmingham Internal Ethics Review Committee (Ref: 2015-6/C1/DK/11) and the Institutional Ethics Committee of Research at the Department of Health, Loreto (Constancia No: 001-CIEI-DRSL-2016).

### Setting

This pragmatic exploratory study was undertaken in the San Juan health centre in Iquitos, north-eastern Peru. The health centre serves 32,848 people in the southern district of the city and offers free state-funded health consultations under the Seguro Integral de Salud (SIS) system for those who qualify as either ‘poor’ or ‘very poor’. Residents who do not qualify for support are still able to access the health centre but a fee is incurred.

### Sampling

The power of a purposive sample lies in the intentional selection of participants defined as ‘information-rich’ [34]. This embraces the importance of individual views to best answer a research question concerning personal perceptions and practices [34]. Participants were purposively selected based on the following eligibility criteria: (i) caregiver to a child under 5 years of age, (ii) aged over 18 years and (iii) living in Iquitos. Following each interview, participant demographics were entered into a database to facilitate exploration of which characteristics (differing in age, gender, level of education and dengue experience) were missing from the sample. Cases with these characteristics were then purposively sought to ensure a maximum variation sample [35].

### Recruitment

Recruitment was undertaken during the ‘wet season’ in February 2016, where the *Aedes aegypti* vector may be more abundant due to higher levels of rainfall [36]. Eligible participants were identified by clinicians and they were then approached by lead author (AF) with support from an interpreter who explained the study and provided participant information sheets. The reason for participant attendance at the health centre was not recorded. It was made clear that participation was voluntary and would not affect the care participants received at the health centre.

### Data collection

Semi-structured interviews followed a topic guide developed using existing, albeit limited, literature [19–25] and discussion within the research team (S1 Topic Guide). The first two interviews were deemed pilots, due to difficulties with the topic guide and translational discrepancies. After this, the topic guide was revised and translation difficulties addressed before the final eighteen interviews. An iterative process allowed inclusion of novel ideas, until no new concepts were presented and data saturation was achieved [37].

Interviews were conducted in English and Spanish using real-time interpretation [38] between the researcher, interpreter and participant. Two interpreters were used throughout the data collection; the first interpreter was present for five interviews and the second present for 15. Both interpreters were English language students at the university in Iquitos, with experience interpreting in clinical environments. Although acknowledged that use of one interpreter is preferable, efforts were made to ensure the similarity between interviews by briefing the second interpreter on the issues arisen from the pilot interviews and thoroughly practising interview questions.

Informed written consent was obtained before each interview which took place in a private room in the health centre. Interviews were audio-recorded and field notes taken. The spoken Spanish was transcribed into English by the researcher and a translator who had experience translating medical projects. Cultural context and the meaning behind colloquialisms were discussed with the researcher to support analysis. A translation lexicon was developed to establish conceptual equivalence [39]. An independent translator reviewed a random sample of two transcripts to assess the quality of translation. Minimal discrepancies between translations were noted, thus no changes were made.

### Data analysis

Data were analysed using Braun and Clarke’s [40] inductive thematic analysis method to identify and interpret themes. An iterative approach was employed, where a constant comparison method ensured data analysis was undertaken in parallel with data collection [41]. Immersion in the data was ensured through repeated reading and familiarisation of the transcripts. Initial codes and themes were generated and a coding rationale produced, with support of NVivo software. Thematic mind maps were developed and themes reviewed using quotes from the text and interviews as a whole.

To improve the credibility of analysis, five of the most data rich transcripts were independently coded by a second analyst (EB). Differences and similarities were evaluated and additional interpretations incorporated into the coding framework. Themes were discussed between the analysts and further reviewed by the research team (LJ, GW) which sought to enrich interpretations and reduce bias presented by a single analyst [42]. A reflexive approach to analysis was employed, where notes were made throughout the analytical process to acknowledge the potential impact of research bias [43]. Continued communication between the lead author (AF) and Peruvian clinicians (GMS) clarified cultural misunderstandings and minimised the risk of cross-cultural bias in interpretation and analysis [39].

## Results

### Participants

Eighteen interviews formed the final analysis dataset, of which equal numbers were male and female. The average age of the sample was 32 years (range 21 to 56 years). Seven participants reported having suffered DF themselves. A demographic summary of the participants is presented in Table 1. The average length of interviews was 36 minutes (range 24–51 minutes).

**Table 1:** Participant demographics.

Participant ID	Age	Gender	Number of children	Travel time to Health Centre (in minutes)	Marital status	Employed?	Occupation	Highest level of education	Personal Dengue experience?
**P3**	27	Female	2	30	Co-habiting	Yes	Baker	High school	No
**P4**	20	Female	1	10	Co-habiting	Yes	Market seller	High school	Yes
**P5**	56	Male	7	5	Married	Yes	Electrician	Higher education	Yes
**P6**	22	Male	1	15	Co-habiting	No	Student	Higher education	No
**P7**	24	Male	2	10	Co-habiting	Yes	Shop-owner	High school	No
**P8**	30	Male	2	40	Married	Yes	Tree cutter	Higher education	Yes
**P9**	28	Female	3	15	Co-habiting	No	N/A	High school	No
**P10**	43	Female	5	15	Co-habiting	No	N/A	Primary	No
**P11**	22	Female	1	5	Single	Yes	Secretary	Higher education	No
**P12**	48	Male	7	10	Co-habiting	Yes	Fisherman	Primary	Yes
**P13**	30	Female	3	10	Co-habiting	No	N/A	Primary	Yes
**P14**	55	Female	4	30	Married	Yes	Primary teacher	Higher education	No
**P15**	45	Male	3	10	Co-habiting	No	N/A	High school	Unsure
**P16**	25	Female	1	3	Co-habiting	No	N/A	High school	No
**P17**	27	Female	2	15	Co-habiting	No	N/A	Primary	Yes
**P18**	22	Male	1	10	Co-habiting	Yes	Policeman	Higher education	No
**P19**	35	Male	1	15	Married	Yes	Business owner	High school	Yes
**P20**	40	Male	2	5	Co-habiting	Yes	Metal worker	Higher education	No

### Findings

**Fig 1:**
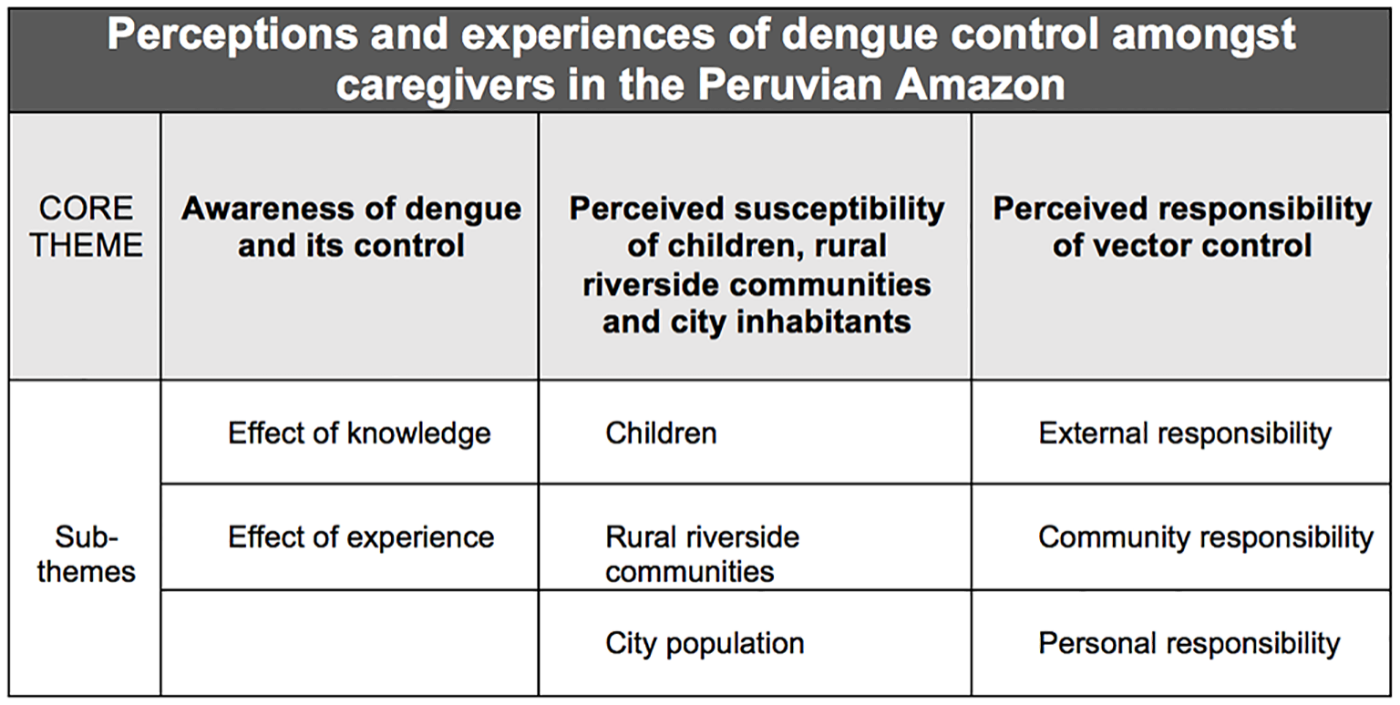
Schematic representation of core analytic themes and subthemes.

Three core analytic themes and eight subthemes (Fig 1) were interpreted within the data: (1) awareness of dengue and its control, (2) perceived susceptibility of children, rural riverside communities and city inhabitants and (3) perceived responsibility of vector control.

### Awareness of dengue and its control

#### Effect of knowledge

All participants had some knowledge about DF as a disease and the ways it can be prevented. Knowledge surrounding general symptoms and mode of transmission was high; all recognised the importance of a persistent “fever”. “Headache” was the next most commonly recognised symptom, then “body pain”, “bleeding”, “chills”, “bone pain” and “rash” respectively. Likewise, all participants knew that the disease was transmitted “by the bite of a mosquito” and some were aware of the process of transmission [P5, P15, P17, P19].

“The transmission of a zancudo* is that it bites an infected person and transfers this to another person.”

[P5]

[*Zancudo is a regional Peruvian name for a type of mosquito which transmits diseases.]

Despite correct knowledge about mosquito transmission, two participants had misconceptions about other ways that the disease can be spread. One participant suggested “*babies can get the disease through breast milk*”, [P18] whilst another suggested that it can arise through drinking “*dirty water*.” [P17]

In terms of effective preventative practices against Dengue, knowledge that focused on removal of mosquito breeding sites was high. The majority of participants recalled the importance of avoiding stagnant water in their homes and backyards through effective cleaning and water container care (all except P8, P15, P16).

“*We are very careful with the water containers… to throw them away, because there can’t be any stagnant water because that is where they breed*.”

[P14]

Others mentioned the use of larvicide provided by health professionals [P3, P5, P6] and household repellent sprays [P4, P6, P17, P20]. Three participants reported wearing “long clothes” as protection [P3, P14, P18]. One participant deviated from the majority and reported: *“I try to keep my home not too dark*.*”* [P16]

However, mosquito biting habits were identified as an error in knowledge in almost all interviews. Only one participant correctly identified that dengue transmitting mosquitoes bite *“during the daytime”* [P15]. Many participants specifically identified that these mosquitoes attacked *“during the night”* [P4, P5, P6, P9, P10, P11, P12, P13, P17] whist other participants were uncertain [P3, P7, P8, P14, P16, P18, P19, P20]. One participant understood that health centre staff had told them so: “*most of the time*, *they [health personnel] tell us that zancudos bite us at night*. *From 5pm*, *yeah I’m sure of this*.” [P11]

Incorrect knowledge appeared to directly affect the way people attempted to protect themselves against mosquitoes. When informed about the actual biting habits, one participant revealed: *“I was doing the wrong thing… because I protect more against mosquitoes in the night rather than in the day*.*”* [P13] In this way, most participants stressed the importance of using mosquito nets at night, some as their primary home protection strategy against Dengue [P8, P12]. Interestingly, the only participant who knew about day-time transmission, also emphasised the need for mosquito nets at night. This demonstrated that even with knowledge of day-time transmission, participants still mistakenly viewed mosquito net use at night as effective prevention behavior:

“*What I know about dengue is that it is a virus (…) that you catch through zancudos, (…) and for that reason you have to use a mosquito net*.”

[P15]

These findings may be explained by the direct conflation (merging of pieces of information) of dengue fever and other diseases such as malaria, which multiple participants demonstrated: *“my son now has Malaria… or Dengue*, *I don’t know which one*!*”* [P8] Often participants could not differentiate between the two diseases in terms of name, symptoms and were unaware that dengue and malaria were transmitted by different mosquitoes with different biting habits.

“*Are Dengue and Malaria the same*?”

[P15]

This conflation demonstrates knowledge inaccuracies associated with Dengue. These participants sought clarification from the interviewer, which highlighted both their lack of knowledge and confidence in the knowledge they possessed.

#### Effect of experience

Often awareness and knowledge about dengue was influenced through direct (i.e. personal) or indirect (i.e. family member or close friend) experience with the disease. Most participants had either direct or indirect experience and were able to recall the signs and symptoms with greater clarity than those who had no experience with the disease.

Seven participants had personally experienced DF (Table 1). Personal experience influenced individual attitude and behaviour towards the disease in two ways. Firstly, it alerted participants to the true severity of the disease: “*it’s not the same when you have a poster stuck on the wall with the symptoms of dengue*, *as having dengue yourself*.*”* [P8] Secondly, it increased interest and participation in preventative practices, through fear of contracting the disease again:

“*Since I have had the disease myself, always and every day I have to clean well, so that my baby can sleep well. I try to stop this disease from happening to me again*.”

[P4]

Likewise, some participants described how dengue experience affected the knowledge, attitudes and interest of other community members. They suggested that the true impact of the disease is better understood when people experience dengue in their families or close friends:

“*When they have dengue, they start worrying about it. Or maybe when a member of their family has dengue, they see that they are ill and what the symptoms are and they start worrying about it by caring for themselves (…) whilst normally, they wouldn’t worry about it*.”

[P3]

### Perceived susceptibility of children, rural riverside communities, city inhabitants

#### Children

The majority of participants highlighted children as a group that were perceived to be most susceptible to DF. Participants offered different explanations to support this view. Some identified the inability of young children to articulate their symptoms as a factor making them more vulnerable to this disease [P4, P9, P10, P11, P14, P20]:

“*Children (…) are more exposed than an adult (…) they cannot talk, they can’t tell us what is happening to them, what is hurting*.”

[P11]

Others suggested that the physical weakness of children influenced their susceptibility [P6, P8, P9, P12]: “*a baby’s body does not have as much resistance as an adult’s*.” [P12]

Two participants explained the vulnerability of children by describing their inability to protect themselves without parental support [P3, P15]:

“*It is more likely that zancudos bite them [children] because they can’t protect themselves if we don’t do anything for them*.”

[P3]

For that reason, there was a strong element of parental concern highlighted, where child protection was identified as a priority for most. One participant in particular stressed the importance of dengue control in order to *“save our lives and the lives of our children*.*”* [P14] Concern was further illustrated through acknowledgement of the potentially fatal nature of DF. This was addressed as a key motivating factor to improve protection:

“*If we don’t take care, we could lose a son or a child*.”

[P14]

#### Rural riverside communities

Multiple participants stressed the perceived greater risk of DF in communities living outside the city in the rural rainforest, referring to it as the ‘jungle’ [P5, P6, P7, P8, P10, P14, P18, P20]. Participants showed concern for these people and saw them as a susceptible group of society in the Amazon.

“*The people that have trouble to recover quickly are those that live in the jungle communities. They are the most vulnerable to catch dengue*.”

[P5]

Some participants spent time working or visiting the rural rainforest, and expressed concern that the people lived there “*without protection*” [P14] due to a perceived higher risk of illness and lack of preventative practices. The vulnerability of rural communities was explored by some through their lack of access to health care services, in contrast to the free and accessible treatment available in the city [P6, P8, P14, P18, P20]:

“*The people in [jungle] communities cure with home remedies using medicinal plants, but it doesn’t work very well. When it is [a] strong [disease], imagine coming to Iquitos from there! Some people die coming here*.”

[P9]

These participants emphasised the significance and danger of living far from health services during an outbreak of dengue. The susceptibility of the rural riverside communities was further acknowledged by one participant who described a sense of ‘rural abandonment’: *“There*, *in the rural zones*, *they are abandoned*. *Here in the city*? *I don’t think so*.*”* [P18] This indicated the lack of attention and resources that people felt were directed towards rural communities.

One participant associated poverty as a factor worsening the susceptibility of rural riverside communities, since poor finances directly affected peoples’ access to health services [P18]:

“*There [rural riverside communities] the people are poor, well not too poor… they just don’t have a lot. For example, someone comes to Iquitos and is bringing their child… they have to come to Iquitos from there to cure them from the disease. You know they don’t have family here, the transport costs and the hotel is going to cost, food costs as well (…) This is a big problem for them*.”

[P18]

#### City population

Generally, participants in the study felt more protected than other groups such as rural riverside communities and children. However, the majority still acknowledged feeling susceptible to dengue due to the perceived inevitability of the disease.

Participants felt susceptible to disease since mosquitoes were perceived to be an invisible threat and thus mosquito bites unavoidable [P6, P8, P9, P10, P11]:

“*You don’t know when the mosquito bites you. For example, right now, a mosquito could bite me and I wouldn’t know if I had Dengue or not*.”

[P6]

Furthermore, the perceived ineffectiveness of preventative practices emphasised the vulnerability that people felt living in Iquitos. One participant voiced: “*we feel protected*, *but we are not*.*”* [P5] Many demonstrated this through the belief that dengue could not be prevented through individual actions alone [P5, P8, P9, P13, P16, P19]. Household prevention seemed futile:

“*As much as you clean your house, the mosquitoes still come in (…) as much as you sleep with a mosquito net, you still notice the bite of mosquitoes on your skin (…) sometimes it is out of our hands to control this*!”

[P5]

In particular, many believed household cleaning [P5, P8], long clothing [P5], garbage collection [P7, P19, P13] and mosquito repellent [P8] was ineffective: “*you can’t prevent that they bite you*. *I use repellent*. *And when you sweat*, *you are unprotected*.*”* [P8] Most participants believed fumigation was an unsuccessful strategy [P3, P4, P5, P6, P7, P8, P9, P11, P13, P17, P19] and some participants attributed this to using a weak poison [P3, P5, P9, P11], or making the “mosquitoes stronger.” [P7]

Additionally, participants mentioned the location of Iquitos in Amazon rainforest as a factor influencing DF. Multiple participants were aware that the climate and heavy rainfall were key in enabling mosquito breeding [P6, P8, P9, P12, P13, P14, P15, P17, P18]. This left participants feeling powerless to prevent the disease: *“As we are in the rural riverside*, *this problem never ends*.*”* [P15]

### Perceived responsibility of vector control

The overarching theme of responsibility runs through each narrative and exists in three interlinked but separate dimensions: (i) external responsibility, (ii) community responsibility, (iii) personal responsibility.

#### External responsibility

The majority of participants believed that the responsibility of dengue prevention lay with external bodies. Some directly acknowledged the government [P14, P20], the health centre [P3, P15, P17] or both [P3, P11, P12, P13] as principally responsible. Others implied more subtly: *“I think that health personnel need to look for other ways to solve this problem*.*”* [P8] The same participant further demonstrated this belief by illustrating frustration and lack of confidence in the authorities.

“*The government plays an important role to prevent it. Maybe the government can give us the health, but they don’t. (…) I wouldn’t be worried about this problem in Iquitos, if the government showed people proper attention*.”

[P8]

In the same way, an undertone of dependence on the health centre was demonstrated in all interviews. The majority highly valued and relied upon the services provided. Most believed that it was the duty of the health centre to provide both protection and treatment against dengue:

“*Here in Iquitos, people need the health personnel to remind them what to do all the time, because this disease is dangerous*.”

[P19]

For other participants, the perception of external responsibility was echoed through their reliance on dengue control services provided by the health centre. For instance, most participants expressed a preference for passively receiving information, rather than actively seeking it. One participant directly acknowledged this by emphasising the duty of the researcher to provide knowledge about preventative practices: *“it’s difficult for some people to protect themselves*, *if* *you* *don’t give knowledge or teach them*.*”* [P20]

In Iquitos, this manifested as a preference for personal home visits by community health workers known colloquially as “charlas” [P3, P9, P10, P11, P16]. These incorporated home inspections for potential mosquito breeding sites and face to face information about dengue:

“*The best method is, is receiving “charlas” (…) a “charla” explains in detail what the announcements don’t tell you. Yeah, you can get all the information in a “charla*.”

[P11]

An additional dengue control service which many participants relied upon was health authority initiated fumigation: *“the most important thing is the fumigation that the regional government do and the authorities*.*”* [P15] Regardless of its perceived efficacy, fumigation was advocated by many [P5, P7, P11, P12, P15, P20]. Some participants questioned its effectiveness but still expressed a need for the practice with ‘stronger poisons’ [P5] or increased frequency [P20]. All participants advocating its use, suggested that fumigation was essential to control the dengue endemic and this further illustrated the belief in external responsibility.

“*I think that they [health personnel] should go to fumigate houses. I think, in that way, we can stop Dengue 100%*.”

[P7]

#### Community responsibility

The majority of participants acknowledged the role of the community as important in dengue prevention. However, this was expressed as a frustration with the lack of community participation in Iquitos. Two participants directly identified the community as principally responsible for dengue prevention [P7, P20]. Many others highlighted an absence of community responsibility through the belief that *“there is not much collaboration from people*.*”* [P19]

Amongst participants’ views, the lack of community “collaboration” took two forms: (i) a lack of concerted effort against mosquito breeding sites and (ii) lack of cooperation with health authorities. The first was described by participants through perceived lack of community adherence to routine larvae surveillance (water container care and larvicide use) and frequent domestic waste build up on the streets: “*some people don’t put their garbage out at the time indicated*, *and zancudos use these places*.*”* [P7] The second aspect to the community problem involved direct inhibition of prevention practices provided by the health centre and was expressed by multiple participants [P3, P4, P7, P17, P19]. *“Some people don’t allow the health personnel to go into their houses”*, [P17] for the purpose of community fumigation, household surveillance or delivery of larvicide and dengue control information.

Participants inferred that there was a community-wide lack of interest and responsibility towards dengue control which worsened the issue. Multiple participants expressed views that other people *“don’t feel interested in it”* [P6] or *“don’t worry about it*.*”* [P3] A further explanation for the lack of community cooperation can be inferred by a perception of community inaction. Many believed that only a small number of community members participated in prevention practices, which acted as a deterrent for other households. Individual household prevention seemed futile if not reciprocated by the entire neighbourhood:

“*It doesn’t work if each family doesn’t clean the water containers properly and maybe your next door neighbour doesn’t do the same as you do and so on, and that can increase Dengue*.”

[P7]

One participant directly acknowledged a solution: *“we need people to collaborate together against this disease*.” [P19] Suggestions were made that the community required “motivation” [P11] to act, in the form of “incentives.” [P19] This same participant suggested providing treated water, rubbish containers or a community information ‘office’ [P19].

#### Personal responsibility

Some participants acknowledged the importance of personal responsibility in dengue prevention. For some, this was expressed as the solitary, most important responsibility [P4, P16, P19]: *“the responsibility is with your own self*.*”* [P4] For others, it was identified as both the responsibility of government or health centre in addition to each individual family [P3, P14, P15, P20].

Some participants expressed the idea through a desire, as caregivers to children under 5 years, to protect themselves and their families from dengue:

“*I want to protect my family (…) I am not going to wait for the government to go to my house*.”

[P16]

For some participants, personal responsibility was shown by an emphasis on family protection: *“who wants to lose someone who you love so much*? *For that reason*, *I had to be more careful with them*.*”* [P19] Additionally, another participant related their reliance on personal responsibility to be due to the failings of the community: *“the community doesn’t do anything when you get ill with dengue*, *because the responsibility is with your own self*.*”* [P4] This feeling was reflected by other participants, who did not feel responsible for anyone other than their own family:

“*The results are for me and not for all my neighbours… because it’s for my own protection*, *for myself and my family*.*”*

[P15]

## Discussion

This pragmatic qualitative study explored the perceptions of the control of dengue fever with 18 Peruvian Amazon caregivers to children under 5 years. Findings highlighted the importance of these perceptions in shaping individual preventative practice in a community. Key findings include the impact of misconceptions around dengue transmission and the importance of attitude within a community for effective preventative practice.

This study found that participants had good basic knowledge of DF symptoms, transmission and eradication of mosquito larvae as preventative practices. In Iquitos, a recent survey found that 65% of people knew someone who had experienced dengue [29]. The current study builds upon this evidence by suggesting explanations for this basic knowledge acquisition such that experience of the disease, information distributed by media campaigns and the existing community health worker system may have influenced and improved knowledge. However, most participants were unaware that dengue mosquitoes bit during the day, which agrees with research by Paz-Soldan *et al*. that identified only 18.6% of respondents recognised the *Aedes aegypti* as a day-biting mosquito and most chose mosquito nets use at night as their main protective practice [29]. The present study further explored the link between these misunderstandings and seeks to partly explain this through a conflation of the information regarding dengue and other mosquito-borne diseases such as malaria, whereby people seem to see these diseases as one combined arbo-viral infection. Perhaps, previous dengue control messages in Iquitos have not provided sufficient information differentiating the two mosquitoes and subsequent diseases. Thus many participants may have perceived malaria and dengue as indifferent, which may have effected their preventative practices. For instance, although effective against malaria prevention, the WHO does not advocate bed nets to prevent dengue fever unless individuals sleep during the day [44]. Thus the specific misconception that mosquitoes transmitting dengue are night-biters directly hindered peoples’ abilities to protect themselves at the correct time.

This study can help to inform health education strategies to be directed towards individuals and communities to build upon basic knowledge, to target misconceptions and make recommendations for successful day-time protection from mosquitoes (such as using household fumigation and insect repellent). Perhaps, health promotion materials need to place more emphasis on distinguishing between dengue and malaria and the biting habits of their associated vectors to better educate and ultimately help the population to protect the themselves. Regular appraisal of the success of these strategies is required to identify and address future misunderstandings. Findings also recommend an evaluation to appraise how the community health worker system influences knowledge and preventative practice.

Furthermore, study findings implied that a hierarchy of risk was perceived by participants. Children and rural riverside communities were felt to be the most vulnerable to DF. A perception and subsequent desire to protect children was echoed in the participants’ responses. It should be acknowledged that these views may be specific to caregivers of children under 5 years, the target group of the study, as reported beliefs could have been affected by social desirability [45]. Dengue control campaigns may wish to target this group by focusing on child protection to incentivise families to participate in preventative strategies. Likewise, rural riverside communities were highlighted as vulnerable groups. In the Loreto region, evidence suggests significantly poorer health outcomes and access to health services in rural communities [46]. Participants proposed differences in mentality between people in rural communities and city as a consequence. A study in the Peruvian Amazon identified a reduced dependence on western medicine in rural communities [47], therefore it may be that these rural communities are forced to take more responsibility for their own health with less access to state provided services. This suggests an area warranting future qualitative research since the health beliefs of these people may require independent dengue control strategies.

Participants viewed themselves as susceptible to dengue since there was a belief that they were unable to protect themselves or their families from this disease. The assumed inevitability of infection, lack of faith in preventative practices and a powerless attitude towards protection compounded this belief. These results are supported by an existing health promotion theory: the Health Belief Model [48]. This model explains how health behaviour is determined by perceptions of a disease and the strategies used to reduce it and has been used an effective tool in previous published research surrounding dengue control [49–52]. In particular, it suggests the impact of perceived susceptibility and self-efficacy as factors influencing health behaviour [48]. In Iquitos, it may be appropriate to suggest that the high perceived susceptibility of dengue positively impacted participants’ preventative practice, whereas a low sense of self efficacy in response to disease prevention may have had the opposite effect. Research in Peru has suggested that a barrier to successful preventative practices may be a perception that control methods like household cleaning are seen as a lot of work. [17] Therefore, acknowledgement that these perceptions shape health behaviour is important for targeting effective health promotion materials.

A heavy reliance on the government or health centre for health protection and education was further illustrated in the study findings and showed parallels with a common concept in healthcare—paternalism [53]. This describes the act of seeking to promote someone’s wellbeing by interfering with their freedom of choice [54]. In some parts of the world, dengue control programmes have been described as “too paternalistic”, focusing on an attitude where the ‘government controls everything’ [55]. In Iquitos, the community health worker “charlas”, mandatory fumigation and household inspections could be viewed in a similar way. Although these services have been linked to a reduction in *Aedes aegypti* indices [56], paternalistic approaches may have the effect of disempowering communities and reinforcing feelings of powerlessness [57]. In turn, disempowered communities may be more susceptible to disease [57].

In this way, the combination of reliance on the government and health centre and a belief that dengue cannot be prevented through individual actions highlights a particular problem with the attitudes of the community in Iquitos, in relation to dengue control. These attitudes highlight a challenge to dengue prevention on an individual level, as well as encouraging a lack of participation and cooperation on a community level and vice versa.

In endemic countries, community participation is vital to ensure the success, suitability and sustainability of dengue control programmes [57]. In this study, a key finding illustrated the frustration that participants felt towards a perceived lack of community participation. Similar findings have been shown in other studies in Latin America where the concept has been described as “*desunión*” or “*mala unión”*. In English this translates to ‘disunion’ and represents a lack of community cooperation in dengue control practices [22,58]. In Ecuador, apathy and social irresponsibility were defined as barriers to community participation [22], the results of which are consistent with the study findings. Although the current study offers explanations behind this social phenomenon, additional qualitative research could add insights and clarity to build upon these findings, perhaps by using a more heterogeneous sample or a focus group design.

Evidence suggests that social mobilization and community efforts are crucial for maintaining dengue prevention [59]. In Iquitos, a lack of community empowerment has been suggested as a barrier to vector control strategies [17]. Thus, there is a need to foster strategies to promote community organisation and empowerment to engage residents in protecting the health of their families and the community as a whole. Successful community-based strategies have been used to reduce the abundance of *Aedes aegypti* in countries such as Cuba, Colombia, Honduras and Mexico [60–63]. For instance in Cuba, ‘community working groups’ were created using existing formal and informal community leaders and took responsibility for the management of vector control strategies in their own constituencies [60]. Other novel approaches have been used such as involving high school students as health educators in Colombia [62] and school-based education in Honduras [63]. Similar strategies could be utilised in Iquitos to encourage a sense of empowerment with campaigns promoting united participation in communities and emphasising the importance of each individual in working towards control of dengue fever. In this way, recommendations may focus on community led groups organising control efforts, perhaps through dissemination of educational materials or promotion of larvae eradication strategies (the Untadita method of cleaning wash basins, for example) [64]. This may prove pertinent since evidence suggests that people in Peru may not be well informed about effective cleaning methods to be able to effectively eliminate the *Aedes aegypti* vector from their homes. [17] Such recommendations seek to improve control of the *Aedes aegypti* vector to not only enhance dengue prevention but protection from other mosquito borne diseases such as zika and chikungunya. Thus, the results from this study may have a wider importance for the public health of this community.

### Strengths and limitations

It is important to acknowledge the strengths and limitations of the study. The strength of this study lies with the important feedback and insight it provides in the ongoing control of dengue fever and the influence of current health campaigns to community and individual perceptions in an endemic region. However, the qualitative nature requires findings to be interpreted with caution, particularly if generalising the conclusions beyond the sample explored. Qualitative studies are limited by the use of a relatively small number of participants and cannot be considered representative [34]. It is important to consider this when applying the findings to caregivers to children under 5 years who live in similar endemic situations.

Furthermore, in cross-language qualitative research, the use of translators and interpreters may impact the findings [39]. Braun and Clarke emphasise the importance of deriving meaning from words and data [65], therefore use of an interpreter and translator may have impacted analysis by increasing the risk that meaning was lost in translation. Steps were taken to avoid this potential bias of misinterpretation [39]. Interviews were piloted, a translation lexicon was created and two transcripts were externally validated. Moreover, the benefits of a local interpreter included useful insights into the meaning behind responses and increased cooperation from participants who seemed more relaxed and willing to participate in in depth discussion with a member of their community.

In retrospect, a further limitation to the study may be the absence of deeper exploration into the conflation presented in the interviews between dengue and other arbo-viral diseases. Further questioning about the different types of mosquitoes known by participants, as well as distinct control methods, may have proved useful when discussing appropriate recommendations to improve vector control. Likewise, the findings from this study would have been further enriched by closer examination of the individual motivators or barriers to performing protective household practices. Further research may aim to better explore these two limitations.

Finally, the impact of the lead author (British, female medical student) conducting cross-cultural research must be acknowledged. Culture is defined as a set of distinctive features of a social group [66] thus, as an outsider to the community, preconceptions and values of the principal researcher may have influenced data interpretation and analysis. The potential effect of this was minimised through support with interpretation from the wider research team, to ensure cultural understanding and the use of multiple coders and analysts to reduce the risk of researcher bias.

### Conclusion

This study contributes to the understanding of perceptions and experiences that influence dengue control amongst caregivers of children under 5 years in an endemic setting. Specific misconceptions around dengue transmission and confusion with other diseases, such as malaria, need to be addressed. In terms of disease control, the lack of community cooperation in mosquito prevention was perceived as a key barrier. This social phenomenon was intensified by attitudes that the government and health centre were responsible for dengue control and the belief that the disease cannot be prevented through individual actions. There is a need to address these perceptions through targeted campaigns encouraging individual and community empowerment in dengue control.

## Supporting information

S1 Topic Guide Topics and questions used to guide qualitative interviews. (DOCX)

## References

[1] World Health Organisation. Dengue guidelines for diagnosis, treatment, prevention and control. Geneva: WHO 2009. 23762963

[2] Farrar J, Focks D, Gubler D, Barrera R, Guzman MG, Simmons C, et al Towards global dengue research agenda. Trop Med Int Health. 2007;12(6):695–699. doi: 10.1111/j.1365-3156.2007.01838.x 17550466 10.1111/j.1365-3156.2007.01838.x PMC4333199

[3] Bhatt S, Gething PW, Brady OJ, Messina JP, Farlow AW, Moyes CL, et al The global distribution and burden of dengue. Nature. 2013; 496(7446): 504–507. doi: 10.1038/nature12060 23563266 10.1038/nature12060 PMC3651993

[4] Halstead SB. The XXth century dengue pandemic: need for surveillance and research. World Health Stat Q. 1992; 45(2–3):292–298. 1462664

[5] San Martin JL, Brathwaite O, Zambrano B, Solórzano JO, Bouckenooghe A, Dayan GH, et al The Epidemiology of dengue in the americas over the last three decades: a worrisome Reality. Am J Trop Med Hyg. 2010;82(1):128–135. doi: 10.4269/ajtmh.2010.09-0346 20065008 10.4269/ajtmh.2010.09-0346 PMC2803522

[6] MINSA. Plan nacional multisectorial e intergubernamental de prevención y control de dengue en el Perú. [cited 2015 Nov 18] http://www.minsa.gob.pe/portalweb/06prevencion/prevencion_2.asp?sub5=5

[7] Gibbons R, Vaughn D. Dengue: an escalating problem. BMJ. 2002;324(7353):1563–6. 12089096 10.1136/bmj.324.7353.1563 PMC1123504

[8] World Health Organisation. Dengue and Severe Dengue. [cited 2015 Nov 6] http://www.who.int/mediacentre/factsheets/fs117/en/

[9] Kularatne S. Clinical Review: Dengue Fever. BMJ. 2015; 351:h4661. 26374064 10.1136/bmj.h4661

[10] Capeding RZ, Brion JD, Caponpon MM, Gibbons RV, Jarman RG, Yoon IK, et al The incidence, characteristics, and presentation of dengue virus infections during infancy. Am J Trop Med Hyg. 2010; 82(2): 330–336. doi: 10.4269/ajtmh.2010.09-0542 20134013 10.4269/ajtmh.2010.09-0542 PMC2813177

[11] Hammond SN, Balmaseda A, Perez L, Tellez Y, Saborío SI, Mercado JC, et al Differences in Dengue Severity in infants, children and adults in a 3 year hospital-based study in Nicaragua. Am J Trop Med Hyg. 2005;73(6):1063–1070. 16354813

[12] World Health Organisation. Comprehensive guidelines for prevention and control of dengue and dengue haemorrhagic fever. WHO SEARO, New Delhi 1999; 23:10–15.

[13] World Health Organisation. Immunization, Vaccines and Biologicals: Dengue vaccine research. [cited 2017 Feb 16] http://www.who.int/immunization/research/development/dengue_vaccines/en/

[14] Capeding MR, Tran NH, Hadinegoro SR, Ismail HI, Chotpitayasunondh T, Chua MN, et al Clinical efficacy and safety of a novel tetravalent dengue vaccine in health children in Asia: a phase 3, randomised, observer-masked, placebo-controlled trial. Lancet. 2014;384(9951):1358–65. doi: 10.1016/S0140-6736(14)61060-6 25018116 10.1016/S0140-6736(14)61060-6

[15] Ministerio de Salud Peru. Casos de dengue por department: Peru 2015. [cited 2015 Nov 18] http://www.app.minsa.gob.pe/bsc/detalle_indbsc.asp?lcind=59&lcobj=4&lcper=1&lcfreg=12/11/2015

[16] Vilcarromero S, Casanova W, Ampuero J, Ramal-Asayag C, Siles C, Díaz G, et al Lecciones aprendidas en el control de aedes aegypti para afrontar el dengue y la emergencia de chikungunya en Iquitos, Perú. Rev Peru Med Exp Salud Publica. 2015;32(1):172–8. 26102121

[17] Organización Mundial de Salud. Documento de sistematización: respuesta a los brotes de dengue en las ciudades de Pucallpa e Iquitos, Perú. Organización Panamericana de la Salud. [cited 2016 May 6] http://apps.who.int/iris/bitstream/10665/173291/1/Sistematizacion-brotes-Iquitos-Pucallpa.pdf

[18] World Health Organization. Sustaining the drive to overcome the global impact of neglected tropical diseases: Second WHO report on neglected tropical diseases. Geneva: WHO; 2013

[19] Phuanukoonnon SBM, Bryan J.H. (2006) Folk knowledge about dengue mosquitoes and contributions of health belief model in dengue control promotion in Northeast Thailand. Acta Trop 99(1):6–14. doi: 10.1016/j.actatropica.2006.05.012 16945318 10.1016/j.actatropica.2006.05.012

[20] Perez-Guerra CL, Zielinski-Gutierrez E, Vargas-Torres D, Clark GG. Community beliefs and practices about dengue in Puerto Rico. Rev Panam Salud Publica. 2009;25(3):218–226. 19454149 10.1590/s1020-49892009000300005

[21] Perez-Guerra CL, Seda H, Garcia-Rivera EJ, Clark GG. Knowledge and attitudes in Puerto Rico concerning dengue prevention. Rev Panam Salud Publica. 2005;17(4):243–53. 15969976 10.1590/s1020-49892005000400005

[22] Ibarra A, Luzadis VA, Cordova M, Silva MJ, Ordoñez T, Beltrán Ayala E, et al A social-ecological analysis of community perceptions of dengue fever and Aedes aegypti in Machala, Ecuador. BMC Public Health. 2014;14:1135 doi: 10.1186/1471-2458-14-1135 25370883 10.1186/1471-2458-14-1135 PMC4240812

[23] Winch P, Lloyd L, Godas MD, Kendall C. Beliefs about the prevention of dengue and other febrile illnesses in Mérida, Mexico. J Trop Med Hyg. 1991;94(6):377–87. 1758008

[24] Arellano C, Castro L, Díaz-Caravantes RE, Ernst KC, Hayden M, Reyes-Castro P. Knowledge and Beliefs about Dengue Transmission and Their Relationship with Prevention Practices in Hermosillo, Sonora. Front Public Health. 2015;3(3):142 26090357 10.3389/fpubh.2015.00142 PMC4453268

[25] Wong L, AbuBakar S. Health beliefs and practices related to dengue fever: a focus group study. PLoS Negl Trop Dis. 2013 7(7): e2310 doi: 10.1371/journal.pntd.0002310 23875045 10.1371/journal.pntd.0002310 PMC3708882

[26] Morrison AC, Minnick SL, Rocha C, Forshey BM, Stoddard ST, Getis A, et al Epidemiology of Dengue Virus in Iquitos, Peru 1999 to 2005: Interepidemic and epidemic patterns of transmission. PLoS Negl Trop Dis. 2010;4(5):e670 doi: 10.1371/journal.pntd.0000670 20454609 10.1371/journal.pntd.0000670 PMC2864256

[27] Hayes CG, Phillips IA, Callahan JD, Griebenow WF, Hyams KC, Wu SJ, et al The epidemiology of dengue virus infection among urban, jungle and rural populations in the Amazon region of Peru. Am J Trop Med Hyg. 1996;55(4):459–63. 8916809 10.4269/ajtmh.1996.55.459

[28] Getis A, Morrison AC, Gray K, Scott TW. Characteristics of the spatial pattern of the dengue vector *Aedes aegypti*, in Iquitos Peru. Am J Trop Med Hyg. 2003;69:494–505. 14695086

[29] Paz-Soldan V, Morrison A, Cordova J et al Dengue Knowledge and Preventive Practices in Iquitos, Peru. Am J Trop Med Hyg. 2015;93(6):1330–7. doi: 10.4269/ajtmh.15-0096 26503276 10.4269/ajtmh.15-0096 PMC4674254

[30] Palma-Pinedo H, Cabrera R, Yagui-Moscoso M. Factores detrás de la renuencia al control vectorial del Dengue en tres distritos del Norte del Perú. Rev Peru Med Exp Salud Publica. 2016; 33(1):13–20 27384618

[31] Guzmán M, García G y Kourí G. El dengue y el dengue hemorrágico: prioridades de investigación. Rev Panam Salud Publica 2006;19(3):204–215. 16640849 10.1590/s1020-49892006000300015

[32] Teixeira M, Costa M, Coelho G, Barreto ML. Recent shift in age pattern of dengue hemorrhagic fever, Brazil. Emerg Infect Dis 2008;14(10):1663 doi: 10.3201/eid1410.071164 18826842 10.3201/eid1410.071164 PMC2609867

[33] Cafferata M, Bardach A, Rey-Ares L. Dengue Epidemiology and Burden of Disease in Latin America and the Caribbean: A Systematic Review of the Literature and Meta-Analysis. Val Health Reg Iss. 2013;2(3):347–356. 10.1016/j.vhri.2013.10.002 29702769

[34] Patton M. Qualitative research and evaluation methods. 3rd ed Thousand Oaks, California Sage Publications: 2002

[35] Suri Harsh. Purposeful Sampling in Qualitative Research Synthesis. Qualitative Research Journal 2011;11(2):63–75.

[36] Stewart Ibarra AM, Ryan SJ, Beltrán E, Mejía R, Silva M, Muñoz Á. Dengue Vector Dynamics (*Aedes aegypti*) Influenced by Climate and Social Factors in Ecuador: Implications for Targeted Control. Mores CN, ed. *PLoS ONE*. 2013;8(11):e78263 doi: 10.1371/journal.pone.0078263 24324542 10.1371/journal.pone.0078263 PMC3855798

[37] DiCicco-Bloom B, Crabtree B. The qualitative research interview. Med Educ. 2006;40(4):314–321. doi: 10.1111/j.1365-2929.2006.02418.x 16573666 10.1111/j.1365-2929.2006.02418.x

[38] Esposito N. From meaning to meaning: the influence of translation techniques on non-English focus group research. Qual Health Res. 2001;11(4):568–79. doi: 10.1177/104973201129119217 11521612 10.1177/104973201129119217

[39] Squires A. Language barriers and qualitative nursing research: methodological considerations. Int Nurs Rev. 2008;55:265–273 doi: 10.1111/j.1466-7657.2008.00652.x 19522941 10.1111/j.1466-7657.2008.00652.x PMC2697452

[40] Braun V and Clarke V. Using thematic analysis in psychology. Qualitative research in psychology. 2006;3(2):77–101

[41] Glaser BG. The constant comparative method of qualitative analysis. Soc Probl 1965; 436–445.

[42] Barbour R. Checklists for improving rigour in qualitative research: a case of the tail wagging the dog? BMJ. 2001;322(7294):1115–1117. 11337448 10.1136/bmj.322.7294.1115 PMC1120242

[43] Primeau L. Reflections on self in qualitative research: stories of family. Am J Occup Ther. 2003;57(1):9–16 12549886 10.5014/ajot.57.1.9

[44] WHO. Dengue control: vector strategies. [cited 2016 Apr 4] http://www.who.int/denguecontrol/control_strategies/en/

[45] Collins M, Shattell M, Thomas S. Problematic interviewee behaviors in qualitative research. West J Nurs Res. 2005;27(2):188–99 doi: 10.1177/0193945904268068 15695576 10.1177/0193945904268068

[46] Martínez A, Villarroel V, Seoane J, del Pozo F. Analysis of information and communication needs in rural primary health care in developing countries. IEEE Trans Inf Technol Biomed. 2005;9(1):66–72 15787009 10.1109/titb.2004.842411

[47] Nawaz H, Rahman M, Graham D. Health risk behaviors and health perceptions in the Peruvian Amazon. Am J Trop Med Hyg. 2001; 65(3):252–6. 11561713 10.4269/ajtmh.2001.65.252

[48] Becker M, Rosenstock I. Compliance with medical advice In Steptoe A, Matthews A. Health care and human behaviour. 1984 London: Academic Press p135–152

[49] Siddiqui TR, Ghazal S, Bibi S, Ahmed W, Sajjad SF (2016) Use of the Health Belief Model for the Assessment of Public Knowledge and Household Preventive Practices in Karachi, Pakistan, a Dengue-Endemic City. PLoS Negl Trop Dis 10(11): e0005129 doi: 10.1371/journal.pntd.0005129 27832074 10.1371/journal.pntd.0005129 PMC5104346

[50] Wong LP, Shakir SMM, Atefi N, AbuBakar S (2015) Factors Affecting Dengue Prevention Practices: Nationwide Survey of the Malaysian Public. PLoS ONE 10(4): e0122890 doi: 10.1371/journal.pone.0122890 25836366 10.1371/journal.pone.0122890 PMC4383514

[51] Wong LP, AbuBakar S, Chinna K (2014) Community Knowledge, Health Beliefs, Practices and Experiences Related to Dengue Fever and Its Association with IgG Seropositivity. PLoS Negl Trop Dis 8(5): e2789 doi: 10.1371/journal.pntd.0002789 24853259 10.1371/journal.pntd.0002789 PMC4031145

[52] Phuanukoonnon S, Brough M, Bryan JH (2006) Folk knowledge about dengue mosquitoes and contributions of health belief model in dengue control promotion in Northeast Thailand. Acta Trop 99(1): 6–14 doi: 10.1016/j.actatropica.2006.05.012 16945318 10.1016/j.actatropica.2006.05.012

[53] Cody W. Paternalism in Nursing and Healthcare: Central Issues and Their Relation to Theory. Nurs Sci Q. 2003; 16(4): 288–296 doi: 10.1177/0894318403257170 14596114 10.1177/0894318403257170

[54] Stanford Encyclopaedia of Philosophy. Public Health Ethics: 2.5 Paternalism. [cited 2016 May 4] http://plato.stanford.edu/entries/publichealth-ethics/#Pat

[55] SciDevNet. Dengue Programmes “too paternalistic”. [cited 2016 May 4] http://www.scidev.net/global/health/news/dengue-programmes-too-paternalistic-.html

[56] Organización mundial de salud. Aprendiendo juntos: sistematización de experiencias sobre control vectorial del dengue en la Amazonia Peruana. Organización Panamericana de Salud. [cited 2016 May 6] http://www.who.int/denguecontrol/Experiencias-control-vectorial-Amazonia-Peruana.pdf

[57] Mitchell-Foster K. Interdisciplinary knowledge translation and evaluation strategies for participatory dengue prevention in Machala, Ecuador. In PhD Thesis. University of British Colombia, Interdisciplinary studies. 2013. [cited 2016 Apr 30] https://open.library.ubc.ca/cIRcle/collections/ubctheses/24/items/1.0076889

[58] Whiteford LM. The ethnoecology of dengue fever. *Med Anthropol* Q. 1997 11; 202–223 9186961 10.1525/maq.1997.11.2.202

[59] Nam V, Kay B, Yen NT. Community mobilization, behaviour change and biological control in the prevention and control of dengue fever in Vietnam. Dengue Bull. 2004; 28:57–61

[60] Sanchez L, Perez D, Cruz G et al Intersectoral coordination, community empowerment and dengue prevention: six years of controlled interventions in Playa Municipality, Havana, Cuba. Tropical Medicine & International Health. 2009; 14:1356–1364 19840350 10.1111/j.1365-3156.2009.02379.x

[61] Lloyd L, Winch P, Ortega-Canto J, Kendall C. Results of a community-based *Aedes aegypti* control programme in Mérida, Yucatan, Mexico. Am J Trop Med Hyg. 1992;46(6):635–642. 1621887 10.4269/ajtmh.1992.46.635

[62] Luna JE, Chain I, Hernández J, Clark GG, Bueno A, Escalante R, et al Social mobilization using strategies of education and communications to prevent dengue fever in Bucaramanga, Colombia. Dengue Bulletin. 2004;28:17–21.

[63] Avila Montes GA, Martinez M, Sherman C, Fernandez Cerna E. Evaluation of an educational module on dengue and Aedes aegypti for schoolchildren in Honduras. Rev Panam Salud Publica.2004;16(2):84–94. 15357933 10.1590/s1020-49892004000800003

[64] Fernández EA, Leontsini E, Sherman C, Chan AS, Reyes CE, Lozano RC et al Trial of a community based behavioural intervention to decrease infestation of Aedes aegypti mosquitoes in cement washbasins in El Progreso, Honduras. Acta Trop. 1998;70(2):171–183 9698263 10.1016/s0001-706x(98)00033-3

[65] Braun V, Clarke V. *Successful Qualitative Research*. London: SAGE Publications Ltd; 2013.

[66] UNESCO. UNESCO Universal Declaration on Cultural Diversity. [cited 2016 Apr 20] http://unesdoc.unesco.org/images/0012/001271/127160m.pdf

