# Supplementary material for: "We need people to collaborate together against this disease": A qualitative exploration of perceptions of dengue fever control in caregivers' of children under 5 years, in the Peruvian Amazon
Source: PLoS Negl Trop Dis. 2017 Sep 5;11(9):e0005755. doi: 10.1371/journal.pntd.0005755 (PMC5600389; doi:10.1371/journal.pntd.0005755)
Supplement: S1 Topic Guide — (DOCX) [file pntd.0005755.s001.docx]

| Topic | Questions and probes | |
| --- | --- | --- |
| DISEASE  &  VECTOR | **DENGUE DISEASE** | |
|  | **Experience** | - Have you ever had Dengue?   - YES: Can you describe to me your experience with the disease?   - NO: Do you know anyone who’s ever had it?     - YES: Has this changed your understanding? How? |
|  | **Knowledge** | - Can you describe to me everything you understand about the disease Dengue Fever?   - Symptoms?   - Do you think you could recognise these symptoms in your young child? Would you look for anything else?   - What difficulties might you have recognising dengue in a young child? - Are you aware/have you ever heard of Dengue Haemorrhagic Fever?   - YES: Can you describe to me what you understand about the disease?     - Consequences of this disease?     - Are you aware of the [higher] **risk** of Haemorrhagic Dengue in young **children**?       - What do you think about this?   - NO: Can you describe to me what you think might happen if Dengue becomes very severe/serious?     - Are you aware of the higher risk of Dengue becoming serious/ life-threatening in young children?       - What do you think about this? |
|  | **Practices** | - Can you describe what you would you do if you thought your child had Dengue? |
|  | **Perceptions** | - Can you describe how **important** you think it is to know about Dengue Fever? Why? - Can describe what **impact** this disease has on your community? |
|  | **MOSQUITO VECTOR** | |
|  | **Knowledge** | - How do you catch Dengue Fever? = Mosquito - Can you describe to me what you know about this mosquito?   - When bites? – NB: DAYTIME - Do you know any other diseases transmitted by this mosquito? (Zika, Chikungunya) |
|  | **Perceptions** | - Can you describe to me the **impact** of mosquitoes in your area?   - How much of a problem are they in your area? Why/Why not? - Can you describe the **importance** of mosquito protection to you and your family? Why? |

| **DENGUE CONTROL** | **HOME** | |
| --- | --- | --- |
|  | **Knowledge** | - Where have you accessed information about Dengue control?   - Methods of information – TV/Radio/Internet?   - How effective do you think these methods are? Which is the most effective way of informing people? |
|  | **Practices** | - Can you describe what you do to stop the Dengue mosquitoes in your home?   - Cleaning/ mosquito nets/ repellent. Anything else? |
|  | **Perceptions** | - How well do you think these practices work? - How well protected do you think your family is in your home? Why? - How important do you think mosquito protection is for homes with young children? |
|  | **Barriers and enablers** | - Is it (sometimes) hard for you to protect your home against mosquitoes? Why? |
|  | **COMMUNITY** | |
|  | **Knowledge** | - How much information do you think your community knows about Dengue and mosquito protection? |
|  | **Practices** | - Can you describe what practices to prevent mosquitoes happen in your community? Anything else? |
|  | **Perceptions** | - In your opinion, is Dengue prevention a government, community or personal problem? Why? - Do you think you/ your community need better information about Dengue/ Mosquito control?   - YES: Any suggestions for improving Dengue control in your area? What would you like to see? |
|  | **Barriers and enablers** | - What are the barriers/difficulties that stop other people from protecting their homes against mosquitoes? - What factors do you think helps people? |
| **Concluding remarks** | **OVERALL:** Can you describe to me your **thoughts** and **feelings** about Dengue Control in IQUITOS?  Is there anything else you think might be important about Dengue Fever that we haven’t talked about? | |
